# Species‐level biodiversity assessment using marine environmental DNA metabarcoding requires protocol optimization and standardization

**DOI:** 10.1002/ece3.4843

**Published:** 2019-01-15

**Authors:** Gert‐Jan Jeunen, Michael Knapp, Hamish G. Spencer, Helen R. Taylor, Miles D. Lamare, Michael Stat, Michael Bunce, Neil J. Gemmell

**Affiliations:** ^1^ Department of Anatomy University of Otago Dunedin New Zealand; ^2^ Department of Zoology University of Otago Dunedin New Zealand; ^3^ Department of Marine Science University of Otago Dunedin New Zealand; ^4^ Trace and Environmental DNA (TrEnD) Laboratory, School of Molecular and Life Sciences Curtin University Perth Western Australia Australia

**Keywords:** biodiversity assessment, eDNA, extraction, filtration, metabarcoding

## Abstract

DNA extraction from environmental samples (environmental DNA; eDNA) for metabarcoding‐based biodiversity studies is gaining popularity as a noninvasive, time‐efficient, and cost‐effective monitoring tool. The potential benefits are promising for marine conservation, as the marine biome is frequently under‐surveyed due to its inaccessibility and the consequent high costs involved. With increasing numbers of eDNA‐related publications have come a wide array of capture and extraction methods. Without visual species confirmation, inconsistent use of laboratory protocols hinders comparability between studies because the efficiency of target DNA isolation may vary. We determined an optimal protocol (capture and extraction) for marine eDNA research based on total DNA yield measurements by comparing commonly employed methods of seawater filtering and DNA isolation. We compared metabarcoding results of both targeted (small taxonomic group with species‐level assignment) and universal (broad taxonomic group with genus/family‐level assignment) approaches obtained from replicates treated with the optimal and a low‐performance capture and extraction protocol to determine the impact of protocol choice and DNA yield on biodiversity detection. Filtration through cellulose‐nitrate membranes and extraction with Qiagen's DNeasy Blood & Tissue Kit outperformed other combinations of capture and extraction methods, showing a ninefold improvement in DNA yield over the poorest performing methods. Use of optimized protocols resulted in a significant increase in OTU and species richness for targeted metabarcoding assays. However, changing protocols made little difference to the OTU and taxon richness obtained using universal metabarcoding assays. Our results demonstrate an increased risk of false‐negative species detection for targeted eDNA approaches when protocols with poor DNA isolation efficacy are employed. Appropriate optimization is therefore essential for eDNA monitoring to remain a powerful, efficient, and relatively cheap method for biodiversity assessments. For seawater, we advocate filtration through cellulose‐nitrate membranes and extraction with Qiagen's DNeasy Blood & Tissue Kit or phenol‐chloroform‐isoamyl for successful implementation of eDNA multi‐marker metabarcoding surveys.

## 1 INTRODUCTION

Environmental DNA (eDNA) metabarcoding is defined as the simultaneous identification of a multitude of species through next‐generation sequencing from environmental samples (soil, sediment, water). eDNA surveys are gaining attention as a novel, noninvasive, time‐efficient, and cost‐effective monitoring method (Thomsen & Willerslev, 2015). Circumventing visual species observation by applying a genetics approach might be especially rewarding to survey the marine biome, where traditional monitoring methods tend to survey only a subset of the community due to time constraints, the inaccessibility of the environment, lack of taxonomic expertise, and funding limitations (Yamamoto et al., 2017). To date, aquatic eDNA has shown great promise as an alternative monitoring method in aquatic environments (Hunter et al., 2015) and in assessing their eukaryotic biodiversity (Stat et al., 2017; Thomsen et al., 2012).

**Figure 1:**
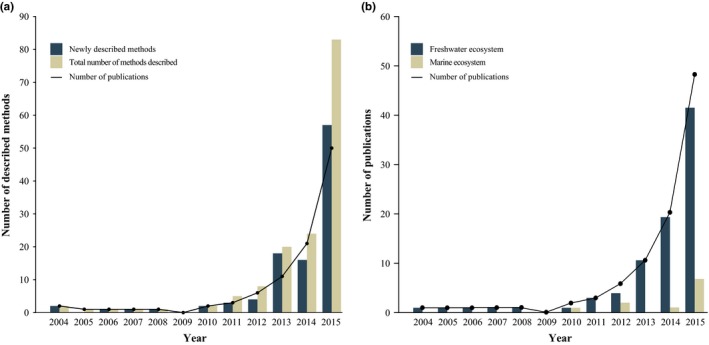
Meta‐analysis on current aquatic eDNA literature displaying (a) the total number of described methods and (b) the proportion of aquatic eDNA research performed in freshwater and marine ecosystems

The recognized potential of eDNA research has led to an exponential increase in eDNA‐related publications (Figure 1). However, a meta‐analysis of the current literature also shows a broadening spectrum of protocols being used to capture and extract eDNA from aquatic samples (Figure 1a; Supplement 1). Prior to qPCR amplification for end‐point analysis, eDNA research follows three general steps. First, a predetermined volume of water is sampled from the habitat of interest. Second, the volume of water is concentrated in a capture step using either precipitation (Dejean et al., 2012), centrifugation (Klymus, Richter, Chapman, & Paukert, 2015), or filtration (Port et al., 2016). Third, DNA is extracted by one of a wide range of commercial kits and modified extraction protocols.

The choice of capture and extraction method is known to significantly influence DNA yields obtained from freshwater samples (Deiner, Walser, Mächlera, & Altermattac, 2015; Eichmiller, Miller, & Sorensen, 2016; Hinlo, Gleeson, Lintermans, & Furlan, 2017; Spens et al., 2016). However, the influence of variation in protocols for the emerging field of marine eDNA is less well established. Recent work suggests that only the capture step in eDNA metabarcoding workflows significantly influences DNA yields from marine samples (Djurhuus et al., 2017). DNA yield in turn affects metabarcoding results in freshwater samples (Deiner et al., 2015), but not in marine samples (Djurhuus et al., 2017). Although findings appear inconsistent between ecosystems, standardization and optimization of capture and extraction methods in eDNA metabarcoding research are desirable to improve comparability between studies (Goldberg, Strickler, & Pilliod, 2015) and the sensitivity of metabarcoding assays.

To date, most eDNA metabarcoding projects, including the recent work on marine eDNA protocol optimization, have used broad‐spectrum single‐marker OTU analyses for biodiversity assessment. However, OTUs are known to overinflate biodiversity estimates in certain cases (Diaz et al., 2012). Additionally, conservation and management decisions still rely on data at the species, rather than the OTU level (Baker et al., 2016; Keith et al., 2015), making biodiversity assessments conducted using OTUs less directly applicable to conservation management. The field of eDNA research is therefore moving toward massive scale species detection through targeted multi‐marker metabarcoding. Although the impact of eDNA capture and extraction protocols on a broad‐scale metabarcoding approach has been determined (Djurhuus et al., 2017), no research has yet been performed to determine the effect on a targeted multi‐marker metabarcoding approach allowing species‐level identification in the marine environment. This information is much needed to maximize the utility of eDNA in marine applications (Figure 1b).

Here, we investigated the performance of the most prevalent capture and extraction protocols for marine water samples using total DNA yield measurements. As filtration is the most commonly used capture protocol for seawater, we evaluated the filtration capture performance for a range of membrane types and pore sizes. We also compared the DNA extraction performance for several extraction protocols. We determined the effect of different capture and extraction protocols on the resulting DNA yield by comparing metabarcoding results from both an optimal and low‐performance capture and extraction protocol. For taxonomic breath, we employed four metabarcoding assays. We assessed OTU and species richness per replicate and the overall detected biodiversity within both treatments for each metabarcoding assay. Our results clearly demonstrate the benefits of optimizing and standardizing eDNA metabarcoding methods prior to widespread implementation in the marine environment.

## 2 MATERIALS AND METHODS

**Figure 2:**
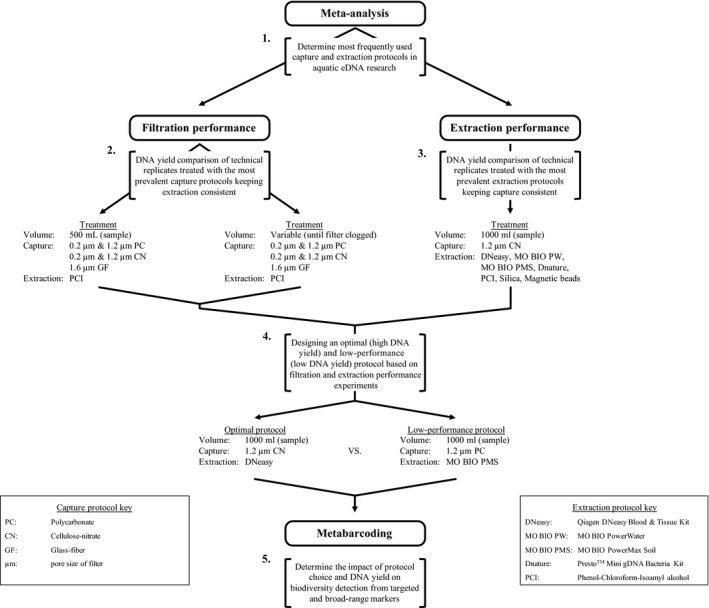
Overview of experimental design

We performed two comparison experiments to assess how multiple capture and extraction protocols affect DNA yield (Figure 2). In the first experiment, we evaluated the capture performance of several commonly employed filter membranes and pore sizes. In the second experiment, we evaluated the performance of the most commonly used extraction protocols according to our meta‐analysis (Supplement 1). From both comparison experiments, we developed an optimal (high DNA yield) and a low‐performance (low DNA yield) protocol. We analyzed metabarcoding data obtained from technical replicates treated with the optimal or low‐performance protocol to determine the effect of protocol choice and DNA yield on multi‐marker eDNA metabarcoding research in the third experiment.

### 2.1 Sample collection and contamination prevention

Prior to field and laboratory work, we sterilized all equipment using a 10‐min exposure to 10% bleach solution (Prince & Andrus, 1992). We decontaminated all sampling bottles (2 L, HDPE Natural, EPI Plastics) by rinsing twice with ultrapure water (UltraPure™ DNAse/RNAse‐Free Distilled Water, Invitrogen™), submerging in 10% bleach for ten minutes, and rinsing twice again with ultrapure water. We checked for contamination at each step in the process. We filled two 2 L bottles with ultrapure water and placed them among the sampling bottles to test for contamination during sampling. We added negative filtration controls by filtering 500 ml ultrapure water and included two extraction blanks consisting of 500 µl ultrapure water. All laboratory work prior to amplification was performed in a PCR‐free designated clean room.

Water samples were collected from Otago Harbor, Dunedin, New Zealand (latitude 45°52′49.33″S, longitude 170°30′22.58″E). We collected surface samples from a pontoon to reduce introducing bias from sediment with higher DNA concentrations mixing with the water column (Turner, Uy, & Everhart, 2015). Samples were collected at the same time for each experiment and transported back to the laboratory within one hour of collection and mixed by inversion to ensure an even distribution of DNA before aliquoting into technical replicates.

### 2.2 Capture performance

We compared capture efficiency of the three most frequently used filter materials: polycarbonate (PC, Millipore™) and cellulose‐nitrate (CN, Whatman™) filters of 0.2 and 1.2 µm pore sizes and a glass‐fiber (GF, Whatman™) filter of 1.6 µm pore size. To compare technical replicates between treatments, we filtered equal volumes of 500 ml for each of the fifty technical replicates, ten for each treatment. After vacuum filtration (Laboport^®^, KNF Neuberger, Inc.), filters were rolled up, cut into ~1 mm slices, placed in 2‐ml Eppendorf tubes, and stored at −20°C until extraction. We opted to treat all samples with a modified phenol‐chloroform‐isoamyl alcohol (PCI) protocol (Renshaw, Olds, Jerde, McVeigh, & Lodge, 2015), ensuring only the capture treatment varied within this first experiment (Supplement 2). PCI was preferred over other protocols, as it minimizes cost and possible spin column contamination (van der Zee et al., 2002). DNA extracts were stored at −20°C until further analysis.

The volume of water able to be processed depends on pore size and filter material. We therefore repeated this experiment by filtering water through each of the five filters until clogging occurred. The volume filtered before clogging was 500 ml for the 0.2‐µm PC filters, 2,500 ml for the 1.2‐µm PC filters, 1,000 ml for the 0.2‐µm CN filters, 3,000 ml for the 1.2‐µm CN filters, and 5,000 ml for the 1.6‐µm GF filters. All samples were extracted with the PCI protocol, and DNA extracts were stored at −20°C until further analysis.

### 2.3 Extraction performance

We compared DNA extraction performance across seven protocols: (a) Qiagen DNeasy Blood & Tissue Kit (Qiagen GmbH, Hilden, Germany), (b) MO BIO PowerWater DNA Isolation Kit (MO BIO Laboratories, Inc., Carlsbad, CA, USA), (c) MO BIO PowerMax Soil DNA Isolation Kit (MO BIO Laboratories, Inc., Carlsbad, CA, USA), (d) Presto™ Mini gDNA Bacteria Kit (Geneaid Biotech Ltd., Taiwan), (e) PCI alcohol DNA extraction protocol (Renshaw et al., 2015), (f) Silica extraction protocol (Alawi, Schneider, & Kallmeyer, 2014; Ogram, Sayler, & Barkay, 1987), and (g) Magnetic Beads extraction protocol (Oberacker et al., 2018). A detailed description of each procedure can be found in Supplement 2. Prior to comparison, each treatment was optimized for DNA extraction from marine water samples. Based on the results from the capture experiment, we sampled seventy 1,000 ml technical replicates, ten for each extraction method. Each sample was vacuum filtered through a 1.2‐µm CN filter. The final elution volume for each DNA extraction protocol was 200 µl to ensure accurate comparison across methods. DNA extracts were stored at −20°C until further analysis.

### 2.4 DNA yield measurement and statistical analysis

Capture and extraction performance were based on total DNA concentration measured in triplicate for each sample on a Qubit^®^ 2.0 Fluorometer (Qubit^®^ dsDNA HS Assay Kit, Invitrogen™). We used a one‐way analysis of variance (ANOVA) to compare capture and extraction performance across the different treatments tested. Post hoc comparisons for ANOVA were performed using Tukey–Kramer's test. All statistical tests were performed in R (http://www.R-project.org).

### 2.5 Metabarcoding

Based on the performance of the different treatments included in the capture and extraction experiments, we designed an optimal (filtration: 1.2 µm CN; extraction: Qiagen's DNeasy Blood & Tissue Kit) and low‐performance (filtration: 1.2 µm PC; extraction: MO BIO's PowerMax Soil DNA Isolation Kit) eDNA protocol. Bigger pore‐sized filters were used to facilitate larger volume processing and to reduce the amount of bacterial DNA captured. We opted to use MO BIO's PowerMax Soil DNA Isolation Kit for extraction in the low‐performance treatment, as it is more frequently used in eDNA research compared to the silica extraction method and magnetic beads according to our meta‐analysis of eDNA literature. We compared technical replicates from both treatments to determine the impact of protocol choice and the subsequent differing DNA yield on the biodiversity detected through metabarcoding. The DNA concentration of twenty 1,000 ml technical replicates, ten for each procedure, was measured in triplicate on Qubit to enable statistical exploration of the difference in DNA yield retrieved between both procedures.

Library preparation followed the protocol described in Ref. (Berry et al., 2017; Stat et al., 2017). Briefly, we chose five random technical replicates per treatment to be analyzed using four metabarcoding assays targeting two fragments of the 16S rRNA gene region, one fragment of the cytochrome c oxidase subunit I (COI) gene region, and one fragment of the 18S rRNA gene region (Table 1 ). Prior to library preparation, input DNA for each sample was optimized for qPCR amplification (StepOnePlus™ Real‐Time PCR System, Thermo Fisher Scientific) (Supplement 3) using a dilution series (neat, 1/5, 1/10). Quantitative PCR amplification of the dilution series with template‐specific primers ensured the determination of inhibitors and low‐template samples. Based on Ct‐value and end‐point fluorescence, the optimal dilution factor for each sample was determined and used during library preparation (Murray, Coghlan, & Bunce, 2015). A one‐step amplification protocol was used for library building using fusion primers, which contained a modified Illumina sequencing adapter, a barcode tag (6–8 bp in length), and the template‐specific primer. Each sample was amplified in duplicate and assigned a unique barcode combination to allow pooling of samples post‐qPCR (Supplement 3 ; Supplement 4). qPCR duplicates of each sample were pooled together to reduce stochastic effects from PCR amplification. Samples were then pooled based on end‐point fluorescence into mini‐pools (of each qPCR). Molarity of mini‐pools was measured on LabChip GX Touch 24 (PerkinElmer, USA). Mini‐pools were combined in equimolar concentrations to produce a single DNA library. The resultant library was size selected using Pippin Prep (size range for both 16S assays: 160–450 bp; size range for COI assay: 200–600 bp) (Sage Science, USA) and purified with Qiagen's QIAquick PCR Purification Kit (Qiagen GmbH, Hilden, Germany) following the manufacturer's protocols. Sequencing was performed on an Illumina MiSeq^®^ (300 cycle single‐end for both targeted assays or 500 cycle paired‐end for both universal assays), following the manufacturer's protocols, with 5% of PhiX to minimize issues associated with low‐complexity libraries.

**Table 1:** Metabarcoding assays and the respective primer sets used for biodiversity detection

Metabarcoding assay	Primer set	Target	Gene	Primer sequence	Amplicon length	References	Assay *T*_m_ (°C)
Fish (16S)	Fish16SF/D	Fish	16S rRNA	5′‐GACCCTATGGAGCTTTAGAC‐3′	~200	Berry et al. (2017)	54
	16S2R			5′‐CGCTGTTATCCCTADRGTAATC‐3′		Deagle *et al.* (2007)	
Crustacean (16S)	Crust16S_F(short)	Crustacea	16S rRNA	5′‐GGGACGATAAGACCCTATA‐3′	~170	Berry et al. (2017)	51
	Crust16S_R(short)			5′‐ATTACGCTGTTATCCCTAAAG‐3′			
Eukaryotes (COI)	mlCOIintF	Metazoa	COI	5′‐GGWACWGGWTGAACWGTWTAYCCYCC‐3′	~313	Leray *et al.* (2013)	51
	jgHCO2198			5′‐TAIACYTCIGGRTGICCRAARAAYCA‐3′		Geller, Meyer, Parker, and Hawk (2013)	
Eukaryotes (18S)	18SF1	Universal	18S rRNA	5′‐GCCAGTAGTCATATGCTTGTCT‐3′	~350	Pochon, Bott, Smith and Wood (2013)	52
	18SR400			5′‐GCCTGCTGCCTTCCTT‐3′			

Forward and reverse reads from the eukaryotes (18S) and eukaryotes (COI) metabarcoding assays were merged using default settings in PEAR v 0.9.10. (Zhang et al., 2014). Reads were separated by barcode and assigned to samples using Geneious v 9.1.6. (Kearse et al., 2012) with no mismatches allowed. Amplicons matching the primer, and sequencing adapter for the single‐end assays, at a 100% level were retained for further analysis. The remaining amplicons were filtered using USEARCH (Edgar, 2010) based on a maximum error of 0.1, minimum length of 100 for single‐end reads and 300 for paired‐end reads, and removal of singleton sequences and sequences containing ambiguous bases. Reads passing quality filtering were clustered at 97% to generate an OTU table, according to standard settings in USEARCH. OTUs with single‐observation occurrence were discarded from the dataset. OTUs passing all quality filtering steps were queried using BLASTn against the full NCBI database.

Taxonomic data for each metabarcoding assay were obtained as follows: (a) all BLAST hits with similarity >97% were retained per OTU, (b) lowest taxonomic level across all remaining BLAST hits for each OTU was used, and (c) species identification required an existing record of the species occurring in New Zealand (Ayling, 1987; Cook & Archer 2010). These criteria led to species identification for both targeted metabarcoding assays, and genus and family identification for the eukaryotes (COI) and eukaryotes (18S) metabarcoding assays, respectively. BLAST hits resulting in unicellular picoplankton and bacteria were discarded, as the focus of this study was on multi‐cellular eukaryotes.

We compared the overall OTU and taxonomic biodiversity retrieved by both optimal and low‐performance treatments, as well as the OTU richness and species richness retrieved per technical replicate for each metabarcoding assay. Student's *t* test was used to compare the difference in OTU and species richness between the optimal and low‐performance eDNA protocol.

## 3 RESULTS

### 3.1 Capture performance

**Figure 3:**
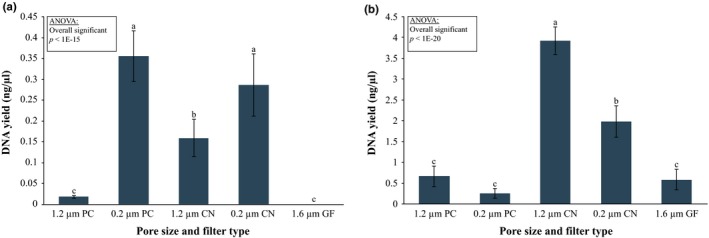
Total DNA yield obtained from five filter membrane types (PC: polycarbonate; CN: cellulose‐nitrate; GF: glass‐fiber) from (a) 500 ml marine water samples and (b) filtration until clogging of the filter. Error bars show 95% confidence intervals. Letters indicate significant differences among groups based on Tukey–Kramer's test (α = 0.05)

Choice of capture method had a significant effect on the total amount of DNA recovered from seawater samples in both experiments according to ANOVA, with *F*_(4,45)_ = 42.901, *p* < 0.0001; *F*_(4,45)_ = 107.693, *p* < 0.0001, for the 500 ml and filtration until clogging experiments, respectively (Figure 3). When filtering small volumes of water, (i.e., 500 ml), filters with smaller pore sizes captured significantly more DNA compared to filters with larger pore sizes, regardless of the membrane type used (Figure 3a). We found no significant (*p* = 0.267) difference between different membrane types with small pore sizes, with an average DNA yield of 0.36 ± 0.06 ng/µl for 0.2‐µm polycarbonate filters and 0.29 ± 0.07 ng/µl for 0.2‐µm cellulose‐nitrate filters. For filters with larger pores, cellulose‐nitrate membranes (average DNA yield: 0.16 ± 0.05 ng/µl) performed significantly better compared to polycarbonate (average DNA yield: 0.02 ± 0.001 ng/µl; *p* = 0.0014) and glass‐fiber (undetectable; *p* = 0.001) membranes.

Membrane type also influenced the maximum volume that could be filtered before clogging, with cellulose‐nitrate filters accepting greater sampling volumes than polycarbonate filters. The increase in sampling volume resulted in a significant increase in mean DNA yield, 3.91 ± 0.33 ng DNA/µl for the 1.2 µm pore size and 2.00 ± 0.37 ng/µl for the 0.2 µm pore size, compared to all other membranes tested (Figure 3b), with the bigger pore size significantly outperforming the smaller pore size according to Tukey–Kramer's post hoc test (*p* = 0.001). There was no significant difference in DNA yield between polycarbonate filter membranes (average of 0.26 ± 0.11 ng/µl for the 0.2 µm pore size and 0.67 ± 0.24 ng/µl for 1.2 µm pore size, *p* = 0.267) and the glass‐fiber membrane (average of 0.58 ± 0.25 ng/µl).

### 3.2 Extraction performance

**Figure 4:**
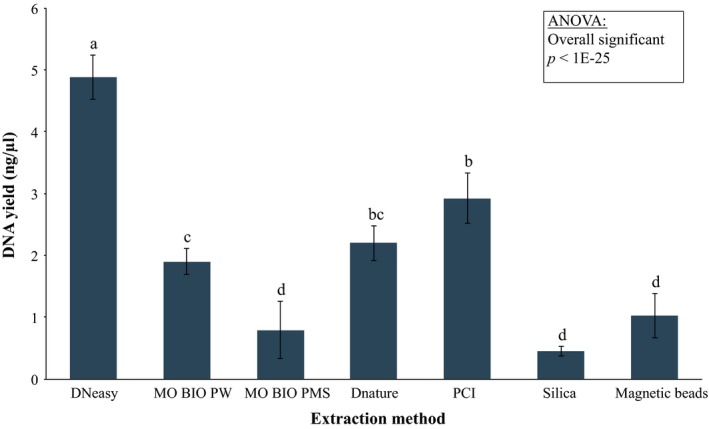
DNA yield from seven extraction protocols (DNeasy: Qiagen DNeasy Blood & Tissue Kit; MO BIO PW: MO BIO PowerWater DNA Isolation Kit; MO BIO PMS: MO BIO PowerMax Soil DNA Isolation Kit; Dnature: Presto™ Mini gDNA Bacteria Kit; PCI: phenol‐chloroform‐isoamyl extraction procedure; Silica: Silica extraction procedure; and Magnetic beads: Magnetic beads extraction procedure) obtained from 1,000 ml technical replicates filtered through 1.2‐µm cellulose‐nitrate filters. Error bars show 95% confidence intervals. Letters indicate significant differences among groups based on Tukey–Kramer's test (α = 0.05)

The choice of extraction method significantly affected the total amount of DNA recovered from 1,000 ml of seawater (*F*_(6,63)_ = 81.83, *p* < 0.0001) (Figure 4). The Qiagen DNeasy Blood & Tissue Kit obtained the highest DNA yield, averaging 4.90 ± 0.35 ng/µl, and significantly outperformed the second‐best performer—the PCI extraction protocol averaging 2.92 ± 0.41 ng/µl (*p* = 0.001). MO BIO's PowerMax Soil DNA Isolation Kit provided the lowest DNA extraction yield with the highest variation between replicates (average DNA yield: 0.79 ± 0.46 ng/µl) compared to all other commercially available kits. We did not detect a significant difference between MO BIO's PowerMax Soil DNA Isolation Kit provided the lowest DNA extraction yield with the highest variation between replicates (average DNA yield: 1.03 ± 0.36 ng/µl, *p* = 0.9), and the least efficient modified DNA extraction protocol, the Silica extraction protocol (average DNA yield: 0.45 ± 0.07 ng/µl, *p* = 0.76), due to the high variation between replicates obtained using the commercial kit.

### 3.3 Metabarcoding approach

Qubit measurements revealed a ninefold increase in DNA yield between the optimal (4.55 ± 0.22 ng/µl) and low‐performance (0.55 ± 0.24 ng/µl) treatment. Importantly, qPCR revealed that across all metabarcoding assays, the target DNA (determined by relative Ct values) from technical replicates generated higher yields than poorly performing methods (Supplement 4). This trend was most obvious for the fish (16S) assay, leading to amplification dropout and reduced species detection for some technical replicates observed with low‐performance methods.

Filtering and quality control returned 2,307,020 reads with 131,592; 226,826; 500,758; and 1,447,844 reads for the fish (16S), crustacean (16S), eukaryotes (COI), and eukaryotes (18S), respectively (Supplement 5). Overall, eDNA samples achieved good sequencing coverage (mean number of reads per sample ± *SD*: fish (16S): 16,449 ±9,978; crustacean (16S): 22,683 ± 7,345; eukaryotes (COI): 50,076 ± 7,656; and eukaryotes (18S): 144,784 ± 28,350). Although PCR products of all negative controls were spiked into libraries to detect contamination, no reads were returned after quality control and filtering. OTU clustering returned a combined total of 1,119 OTUs with 8; 31; 452; and 628 OTUs for the fish (16S), crustacean (16S), eukaryotes (COI), and Eukaryotes (18S), respectively. BLAST retained a total of 85 taxa with 6 (75.0%), 14 (45.2%), 16 (3.5%), and 49 (7.8%) taxa for the fish (16S), crustacean (16S), eukaryotes (COI), and eukaryotes (18S), respectively (Supplement 6).

**Figure 5:**
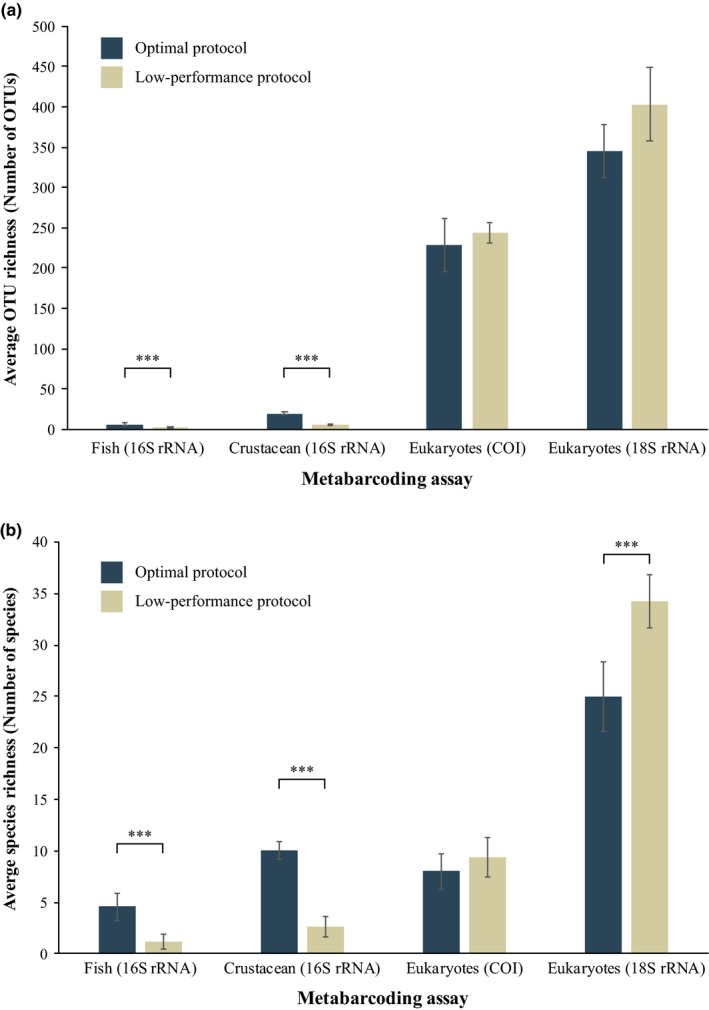
The average (a) OTU and (b) taxon richness obtained per replicate for each of the four assays between the optimal (blue; 1.2‐µm cellulose‐nitrate filter and Qiagen's DNeasy Blood & Tissue Kit) and low‐performance (gold; 1.2‐µm polycarbonate filter and MO BIO's PowerMax Soil) protocol. Error bars show 95% confidence intervals. *T* test significance is depicted by: *** *p* < 0.001

**Figure 6:**
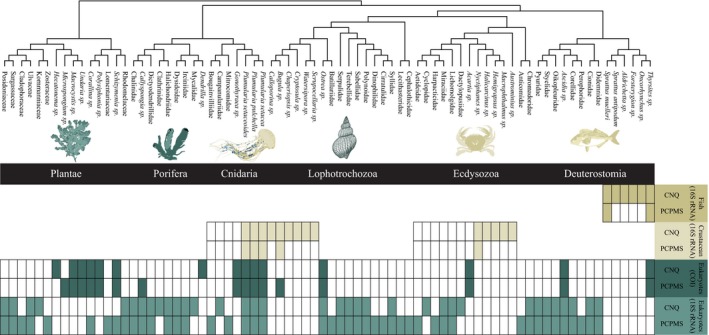
Observed taxa in each of the four assays. Filled and unfilled rectangles indicate taxon presence or absence. Taxa show a pair of rectangles per assay, representing the low‐performance (PCPMS; 1.2‐µm polycarbonate filter and MO BIO's PowerMax Soil) and the optimal (CNQ; 1.2‐µm cellulose‐nitrate filter and Qiagen's DNeasy Blood & Tissue Kit) treatment. Rectangular presence indicates the amplification range of each assay

Species and OTU detection from targeted fish and crustacean assays were strongly affected by protocol choice and the differing DNA yield. The increased total and target DNA obtained using the optimal protocol resulted in a ~5‐fold increase in average OTU and species richness per replicate for both assays (Figure 5). Besides increased richness, increased DNA yields also resulted in more consistent OTU and species detection across replicates (Supplement 6). For both assays, many species were left undetected when the low‐performance protocol was used, with six versus two fish; and fourteen versus five crustacean species detected for the optimal and low‐performance protocols, respectively (Figure 6; Supplement 6). In contrast to the targeted assays, protocol choice and the differing DNA yield had little effect on either OTU and taxon richness detected via the broad‐scale eukaryotic metabarcoding assays (Figure 5), with the opposite trend showing increased richness for replicates treated with the low‐performance protocol only being significant for species richness of the eukaryotes (18S) assay.

## 4 DISCUSSION

Our results demonstrate that protocol choice in both the capture and extraction step in eDNA experiments significantly affects the DNA yield obtained from marine samples. We found that optimization of both steps led to a ninefold increase in total DNA yield. In addition, we reveal that replicates with higher DNA yields produce increased species richness detection and improved consistency between replicates for targeted metabarcoding assays. Our data, therefore, illustrate the importance of protocol optimization and the need for consistent protocol use to facilitate successful implementation of targeted multi‐marker eDNA metabarcoding surveys and ensure comparability between studies.

### 4.1 Capture performance

Processing aquatic samples in eDNA studies starts with reducing the volume and capturing DNA by filtration, for which membrane type and pore size of the filter vary between studies (Supplement 1). Membranes with small pore sizes (0.2 µm) produced an increased DNA yield, irrespective of the membrane type used for the same filtered volume of water. However, total DNA yield does not distinguish between eukaryotic target DNA and bacterial DNA. Bacteria are capable of passing through the 1.2 and 1.6 µm pore sizes (Turner, Miller, Coyne, & Corush, 2014); thus, the increased DNA yield seen when 0.2‐µm filters were used could be attributed to more microorganisms being captured by that pore size, but not others (Stat et al., 2017). If true, this would render comparisons between different pore sizes invalid. Taking this into account, although we did not a detect a difference in DNA yield between the two membranes with 0.2 µm pores, cellulose‐nitrate filters with 1.2 µm pores significantly increased DNA yield compared to other filters with larger pore sizes tested.

Sample volume usually exceeds the limit of filters with smaller pores (Uchii, Doi, & Minamoto, 2016); thus, we also filtered the maximum volume possible to determine volume limit and maximum DNA yield for each filter. In this case, cellulose‐nitrate filters outperformed polycarbonate filters of the same pore size both in maximum volume filtered and in DNA yield. Although the glass‐fiber filter tested in this study had the largest pore size and allowed the largest sampling volume to pass through, DNA yields for this filter were significantly lower than cellulose‐nitrate membranes, as has been found in other studies (Djurhuus et al., 2017).

Filtration was the sole method tested in this study, as it is currently the most prevalent capture method in aquatic eDNA research. Previous research performed in freshwater ecosystems (Deiner et al., 2015; Eichmiller et al., 2016; Hinlo et al., 2017) shows filtration outperforms both centrifugation and precipitation. We recommend using cellulose‐nitrate filtration to capture DNA in future eDNA studies conducted in temperate marine environments, a finding in agreement with that for the freshwater ecosystem (Hinlo et al., 2017). These findings are suggesting some commonalities across aquatic eDNA sampling environments. Choice of filter pore size for a given study will be dependent on the number of suspended particles and the turbidity of the water being sampled (Table 2). Recently, the Sterivex™ filtration system has gained popularity and was found to be the optimal capture strategy in freshwater eDNA research (Spens et al., 2016). In Sterivex™ systems, the plastic housing surrounding the filter circumvents the need for a vacuum filtration manifold, allowing for in‐field filtration strategies. The faster processing time of samples might aid in halting eDNA degradation until extraction. However, the significant increase in cost per filter might make Sterivex™ filters less desirable in some applications. Comparative studies in the marine environment between the Sterivex™ filtration system and vacuum filtration are needed to determine the advantages and drawbacks of both capture protocols.

**Table 2:** Recommendation and critical considerations of laboratory protocol for future marine eDNA research

	Volume	Membrane type	Pore size	Extraction method	Metabarcoding approach
Recommendation	Maximum possible volume	Cellulose‐nitrate	1.2 µm	Qiagen's DNeasy Blood & Tissue Kit	Multiple targeted metabarcoding assays informing about species of interest
Considerations	Dependent on sampling site and turbidity of water	Similar finding in freshwater ecosystem. Result obtained by comparing most prevalent membranes in eDNA research. Future work might want to compare membrane types less frequently used	Pore size recommended for sample volume >1,000 ml or highly turbid water. 0.2‐µm pore‐sized filters are recommended for small sampling volumes (<1,000 ml) of pristine water	We strongly suggest using the phenol‐chloroform‐isoamyl extraction procedure to reduce cost and contamination risk	Targeted assays amplify only a subset of the biodiversity present in an eDNA sample but allow species identification and obtain consistent results. Universal assays can be used to give a general overview of the biodiversity

### 4.2 Extraction performance

A myriad of commercial kits and adapted extraction protocols are currently used for eDNA research. A DNA extraction method should ideally retain high DNA yields while successfully removing PCR inhibitors. Overall, Qiagen's DNeasy Blood & Tissue Kit generated the highest DNA yield. These findings are consistent with recent studies on freshwater eDNA protocols (Deiner et al., 2015; Hinlo et al., 2017) and further support commonalities between both the freshwater and marine aquatic system. However, spin columns in commercial extraction kits are often contaminated (van der Zee et al., 2002), which could not only lead to an overestimation of DNA extracted from the sample, but also interfere with metabarcoding. Factoring in the cost per sample, one might prefer the modified phenol–chloroform extraction method (Renshaw et al., 2015), which retained the second‐highest DNA yield and significantly outperformed the remaining extraction methods. However, the necessity of a fume hood for phenol–chloroform extractions and the development of automated sample preparation (QIAcube) to enable high‐volume sample processing might increase the suitability of Qiagen's DNeasy Blood & Tissue Kit in processing eDNA samples in certain cases (Table 2).

### 4.3 Metabarcoding approach

Optimizing the capture and extraction step in our marine eDNA protocol resulted in a ninefold difference in total DNA yield compared to the poorest performing methods. Although total DNA yield is not necessarily indicative of target DNA yield (Stat et al., 2017), lower Ct‐values were recorded with increased total DNA yield for all four metabarcoding assays tested in this study (Supplement 4). Protocol choice, which resulted in differing total DNA yield and starting template, significantly affected downstream metabarcoding analyses. Unfortunately, due to the inherent connection, we were unable to determine whether the protocol used, or the obtained DNA yield had a bigger influence. However, both DNA yield (Murray et al., 2015; Saulnier, Decker, & Haffner, 2009), through intensified stochasticity in early PCR amplification cycles, and the protocol used (Eichmiller et al., 2016; Hinlo et al., 2017), through the introduction and removal of specific contaminants and inhibitors, are known to affect downstream metabarcoding analyses.

Neither of the broad‐scale metabarcoding assays tested (COI and 18S) displayed a difference in the average OTU richness per replicate between the optimal and low‐performance protocol. This result is in agreement with the only other evaluation of marine eDNA processing protocols (Djurhuus et al., 2017). The average family richness for the eukaryotes (18S) assay per replicate, however, did show a significant difference in favor of the low‐performance protocol. Despite a lack of impact in these broad‐scale metabarcoding assays, a highly variable metabarcoding output was retrieved between replicates within each treatment and between treatments. The variability in metabarcoding results led to differences in the overall biodiversity retrieved between the optimal and low‐performance protocol (Supplement 6).

Environmental samples contain a highly complex eDNA signal, due to the presence of DNA signatures from the entire community. The enormous complexity is likely linked to the variability seen in our broad‐scale metabarcoding assays and illustrates the difficulties of using universal primer sets to assess the true biodiversity in complex eDNA samples, such as those commonly sourced from marine systems. More intensive sampling and deeper sequencing would be required to accurately represent the biodiversity using universal primers, both of which would add time and financial costs to any study. Although we note that sequencing costs continue to decrease, as the technology advances. Many universal primer sets are also unable to generate species‐level assignments in metabarcoding studies, due to incomplete reference datasets and current sequencing constraints (Soergel, Dey, Knight, & Brenner, 2012). Thus, currently, some universal metabarcoding assays result in low taxonomic resolution. OTU analyses, which make up the bulk of eDNA metabarcoding research to date, have been proposed to alleviate some of the problems associated with universal primer sets (Yoon et al., 2016). However, OTU metrics are known to inflate biodiversity estimates (Diaz et al., 2012). Moreover, OTU analyses do not provide species‐level information that may be required in certain instances of ecosystem conservation (Baker et al., 2016; Keith et al., 2015). The field of eDNA research is therefore moving toward species detection at massive scale through targeted multi‐marker metabarcoding, making the performance of targeted assays much more important to the future of the discipline.

While targeted assays may be more relevant for biodiversity surveys, they are also more sensitive to protocol choice. The increased DNA yield obtained through optimization of the capture and extraction step led to higher OTU and species richness estimates for both targeted assays. Our results clearly show, for the first time, that targeted metabarcoding assays amplifying low‐abundance taxonomic groups (i.e., fish and crustaceans) are far more sensitive to protocol choice and the resulting reduced DNA yields compared to broad‐scale metabarcoding assays. Stochasticity in the early cycles of qPCR amplification, intensified in low copy number situations (Saulnier et al., 2009), likely reduced the number of detected species and the consistency between replicates when the low‐performance protocol was used. Most affected was the fish (16S) assay, which led to amplification issues and inconsistent detection results between replicates with lower DNA yields. Multiple species known to occur in the Otago Harbour (Ayling, 1987) were only detected when the optimal capture and extraction methods were used (e.g., New Zealand blueback sprat (*Sprattus antipodum*); yellow‐eye mullet (*Aldrichetta forsteri*); chinook salmon (*Oncorhynchus tshwaytscha*); and common triplefin (*Forsterygion lapillum*)). A similar pattern was seen in the crustacean (16S) assay, where several species from the infraorder Brachyura (crabs), a group frequently used in monitoring surveys (van Oosterom, King, Negri, Humphrey, & Mondon, 2010), were not detected when the low‐performance protocol was used (e.g., common rock crab (*Hemigrapsus sexdentatus*); stalk‐eyed mud crab (*Macrophthalmus hirtipes*); and variable pillbox crab (*Halicarcinus varius*)).

## 5 CONCLUSION

Targeted metabarcoding assays amplifying low‐abundance taxonomic groups appear more affected by protocol choice and the resulting DNA yield difference than broad‐scale metabarcoding assays. A shift toward assays allowing species identification in eDNA metabarcoding studies, therefore, requires optimization of eDNA capture and extraction protocols prior to commencing marine eDNA studies to reduce cost and to increase efficiency and applicability. Our results suggest that capture via cellulose‐nitrate membranes and extraction via Qiagen's DNeasy Blood & Tissue Kit present an optimal protocol, but that phenol–chloroform extraction may be preferable to reduce cost and lower contamination risk depending on the circumstances. Targeted metabarcoding, after optimization and standardization, has the potential to address the challenges of assessing and monitoring biodiversity in the vast and inaccessible marine biome. Using our optimal capture and extraction protocol, we obtained data on 20 species, 16 genera, and 49 families in a time‐efficient and cost‐effective manner, clearly illustrating the power and potential of aquatic eDNA monitoring in the marine environment when correctly optimized.

## CONFLICT OF INTEREST

None declared.

## AUTHOR CONTRIBUTION

Gert‐Jan Jeunen: This person made substantial contributions to the conception, acquisition, analysis, and interpretation of the data. This person also made substantial contributions in drafting the manuscript. This person gave final approval of the version to be published. This person agreed to be accountable for all aspects of the work; Michael Knapp: This person made substantial contributions to the conception, acquisition, analysis, and interpretation of the data. This person also made substantial contributions in drafting the manuscript. This person gave final approval of the version to be published. This person agreed to be accountable for all aspects of the work; Hamish G. Spencer: This person made substantial contributions to the conception, acquisition, analysis, and interpretation of the data. This person also made substantial contributions in drafting the manuscript. This person gave final approval of the version to be published. This person agreed to be accountable for all aspects of the work; Helen Taylor: This person made substantial contributions to the conception, acquisition, analysis, and interpretation of the data. This person also made substantial contributions in drafting the manuscript. This person gave final approval of the version to be published. This person agreed to be accountable for all aspects of the work; Miles D. Lamare: This person made substantial contributions to the conception, acquisition, analysis, and interpretation of the data. This person also made substantial contributions in drafting the manuscript. This person gave final approval of the version to be published. This person agreed to be accountable for all aspects of the work; Michael Stat: This person made substantial contributions to the conception, acquisition, analysis, and interpretation of the data. This person also made substantial contributions in drafting the manuscript. This person gave final approval of the version to be published. This person agreed to be accountable for all aspects of the work; Michael Bunce: This person made substantial contributions to the conception, acquisition, analysis, and interpretation of the data. This person also made substantial contributions in drafting the manuscript. This person gave final approval of the version to be published. This person agreed to be accountable for all aspects of the work; Neil J. Gemmell: This person made substantial contributions to the conception, acquisition, analysis, and interpretation of the data. This person also made substantial contributions in drafting the manuscript. This person gave final approval of the version to be published. This person agreed to be accountable for all aspects of the work.

## Data Availability

All sequence data and bioinformatics scripts will be archived on Sequence Read Archive (SRA) upon acceptance of the paper.
