# Supplementary material for: Species‐level biodiversity assessment using marine environmental DNA metabarcoding requires protocol optimization and standardization
Source: Ecol Evol. 2019 Jan 15;9(3):1323–35. doi: 10.1002/ece3.4843 (PMC6374651; doi:10.1002/ece3.4843)
Supplement: Supplementary file 2 [file ECE3-9-1323-s002.docx]

Supplement 2: Protocol description.

*Qiagen DNeasy Blood & Tissue Kit*:

- Roll up filters and cut into ca. 1 mm slices and place in 2 mL tubes.
- Add 0.5 g of 0.5 mm Zirconia/Silica Beads and 800 μL ATL buffer.
- Vortex at maximum speed for 1 minute.
- Incubate samples at 56°C for 30 minutes.
- Vortex at maximum speed for 1 minute.
- Spin down for 1 minute at 6000 x g.
- Transfer supernatant (600 μL) into a new 2 mL tube.
- Add 600 μL AL buffer.
- Vortex at maximum speed for 1 minute.
- Incubate samples at 56°C for 10 minutes.
- Add 600 μL ethanol and mix thoroughly by vortexing.
- Pipet the mixture into a DNeasy Mini spin column placed in a 2 mL collection tube.
- Centrifuge at 6000 x g for 1 minute.
- Discard the flow through and collection tube.
- Place the spin column in a new 2 mL collection tube and add 500 μL Buffer AW1.
- Centrifuge samples for 1 minute at 6000 x g.
- Discard the flow through and collection tube.
- Place the spin column in a new 2 mL collection tube and add 500 μL Buffer AW2.
- Centrifuge samples for 3 minutes at 20000 x g.
- Discard the flow through and collection tube.
- Transfer the spin column in a 2 mL centrifuge tube.
- Elute DNA by adding 2 x 100 μL AE buffer to the center of the membrane.
- Incubate for 1 minute at room temperature.
- Centrifuge samples for 1 minute at 6000 x g.
- Store DNA at -20°C.

*MO BIO PowerWater DNA Isolation Kit*:

- Insert the filter into a 5 mL PowerWater Bead tube.
- Add 1 mL of Solution PW1 to the PowerWater Bead tube.
- Vortex the PowerWater Bead tube at maximum speed for 5 minutes.
- Centrifuge tubes at 4000 x g for 1 minute.
- Transfer the supernatant to a clean 2 mL Collection tube.
- Centrifuge samples at 13000 x g for 1 minute.
- Avoiding the pellet, transfer the supernatant to a clean 2 mL Collection tube.
- Add 200 μL of Solution PW2 and vortex briefly to mix.
- Incubate at 4°C for 5 minutes.
- Centrifuge samples at 13000 x g for 1 minute.
- Avoiding the pellet, transfer the supernatant to a clean 2 mL Collection tube.
- Add 650 μL of Solution PW3 and vortex briefly to mix.
- Load 650 μL of supernatant onto a Spin Filter.
- Centrifuge samples at 13000 x g for 1 minute.
- Discard the flow through.
- Repeat until all the supernatant has been loaded onto the Spin Filter.
- Place the Spin Filter basket into a clean 2 mL Collection tube.
- Shake to mix Solution PW4 before use.
- Add 650 μL of Solution PW4.
- Centrifuge samples at 13000 x g for 1 minute.
- Discard the flow through.
- Add 650 μL of Solution PW5.
- Centrifuge samples at 13000 x g for 1 minute.
- Discard the flow through.
- Centrifuge samples again at 13000 x g for 2 minutes to remove residual wash.
- Place the Spin Filter basket into a clean 2 mL Collection tube.
- Add 200 μL of Solution PW6 to the center of the white filter membrane.
- Centrifuge samples at 13000 x g for 1 minute.
- Discard the Spin Filter basket.
- Store DNA at -20°C.

*MO BIO PowerMax Soil DNA Isolation Kit*:

- Insert the filter into a Bead tube.
- Add 15 mL of Powerbead Solution to the Bead tube.
- Vortex at maximum speed for 1 minute to mix.
- Add 1.2 mL of Solution C1.
- Vortex at maximum speed for 10 minutes.
- Centrifuge samples at 2500 x g for 3 minutes.
- Transfer the supernatant to a clean Collection tube.
- Add 5 mL of Solution C2 to the supernatant and invert twice to mix.
- Incubate at 4°C for 10 minutes.
- Centrifuge samples at 2500 x g for 4 minutes.
- Avoiding the pellet, transfer the supernatant to a clean Collection tube.
- Add 4 mL of Solution C3 to the supernatant and invert twice to mix.
- Incubate at 4°C for 10 minutes.
- Centrifuge samples at 2500 x g for 4 minutes.
- Avoiding the pellet, transfer the supernatant to a clean Collection tube.
- Shake to mix Solution C4.
- Add 30 mL of Solution C4 to the supernatant and invert twice.
- Fill the Spin Filter with solution from previous step.
- Centrifuge at 2500 x g for 2 minutes.
- Discard the flow through.
- Repeat process until the entire volume has been processed.
- Add 10 mL of Solution C5 to the Spin Filter.
- Centrifuge at 2500 x g for 3 minutes.
- Discard the flow through.
- Centrifuge the Spin Filter at 2500 x g for 5 minutes.
- Carefully place the Spin Filter in a new Collection tube.
- Add 5 mL of Solution C6 to the center of the Spin Filter membrane.
- Centrifuge at 2500 x g for 3 minutes.
- Discard the Spin Filter.
- Add 0.2 mL of 5 M NaCl and invert 5 times to mix.
- Add 10.4 mL of 100% ice-cold ethanol and invert 5 times to mix.
- Centrifuge at 2500 x g for 30 minutes.
- Decant all liquid and air dry residual ethanol.
- Resuspend precipitated DNA in 200 μL of sterile water.
- Store DNA at -20°C.

*Presto^TM^ Mini gDNA Bacteria Kit*:

- Place filter in a 5 mL tube.
- Add 0.5 g of 0.5 mm Zirconia/Silica Beads.
- Add 1 mL Plant GeneZOL reagent.
- Vortex samples at maximum speed for 5 minutes.
- Transfer 1 mL of supernatant to a new 2 mL tube.
- Incubate samples at 65°C for 15 minutes.
- Centrifuge samples at 15000 x g for 5 minutes.
- Transfer the supernatant to a new 2 mL tube.
- Add 600 μL of Chloroform.
- Vortex samples at maximum speed for 5 seconds.
- Centrifuge samples at 15000 x g for 5 minutes.
- Carefully remove the upper layer and transfer to a new 2 mL tube (800 μL).
- Add 800 μL of Isopropanol.
- Mix samples by gently inverting 20 times. Let stand for 5 minutes.
- Centrifuge samples at 15000 x g for 20 minutes to form a tight, well-formed DNA pellet.
- Carefully remove the supernatant.
- Add 1 mL of 100% ethanol to the DNA pellet and wash by gently inverting 20 times.
- Centrifuge samples at 15000 x g for 3 minutes.
- Carefully remove supernatant, air-dry pellet for 10-15 minutes.
- Resuspend pellet in 50 μL ddH_2_O.
- Add 50 μL CLEAN buffer and mix well.
- Incubate in fridge for 15 minutes.
- Centrifuge at 15000 x g for 1 minute and transfer supernatant to new 2 mL tube.
- Add 100 μL GT buffer and 200 μL GB buffer.
- Vortex samples and pulse spin.
- Add 200 μL 100% ethanol.
- Transfer the sample into a 2 mL spin column.
- Centrifuge at 15000 x g for 30 seconds and discard the flow-through.
- Place the spin column in another 2 mL collection tube.
- Add 600 μL Wash buffer into the center of the spin column.
- Centrifuge at 15000 x g for 30 seconds, discard flow-through, and place in new 2 mL tube.
- Centrifuge again for 3 minutes at 15000 x g to dry the column matrix.
- Transfer the dried column to a new 2 mL tube.
- Add 200 μL of pre-heated elution buffer (60°C) into the center of the column matrix.
- Incubate the column for 2 minutes on block at 60°C.
- Centrifuge for 30 seconds at 15000 x g to elute the purified DNA.
- Store DNA at -20°C.

*Phenol-Chloroform-Isoamyl extraction procedure*:

- Place filter in a 2 mL centrifuge tube.
- Add 900 μL CTAB buffer.
- Vortex samples at maximum speed for 5 seconds.
- Incubate samples for 10 minutes at 65°C.
- Add 900 μL PCI.
- Vortex samples at maximum speed for 5 seconds.
- Centrifuge for 5 minutes at 15000 x g.
- Transfer 700 μL of the aqueous layer into a new 2 mL centrifuge tube.
- Add 700 μL CI.
- Vortex samples at maximum speed for 5 seconds.
- Centrifuge for 5 minutes at 15000 x g.
- Transfer 500 μL of the aqueous layer into a new 2 mL centrifuge tube.
- Add 1.250 mL ice-cold 100% ethanol.
- Add 20 μL of 5 M NaCl.
- Precipitate samples at -20°C overnight.
- Centrifuge samples for 10 minutes at 15000 x g.
- Discard the supernatant.
- Air-dry pellet until no visible liquid remains.
- Rehydrate pellet in 200 μL ultrapure water.
- Store DNA at -20°C.

*Silica extraction procedure*:

- Place filter in a 50 mL tube.
- Add 20 mL of GuHCl buffer.
- Add 2 g of 0.5 mm Zirconia/Silica Beads.
- Vortex samples at maximum speed for 1 minutes.
- Centrifuge samples at 6084 x g for 30 minutes and recover supernatant.
- Add 100 μL of silica suspension.
- Place vials on a horizontal shaker at 150 shakes per minute for 60 minutes.
- Let samples settle for 3 minutes.
- Centrifuge for 3 minutes at 10000 x g at room temperature.
- Discard the supernatant.
- Clean pellet by resuspending the sample in 1 mL washing buffer.
- Centrifuge for 3 minutes at 10000 x g to remove supernatant.
- Air-dry samples for 15 minutes.
- Resuspend pellet in 200 μL dH_2_O.
- Incubate for 5 minutes at 50°C.
- Centrifuge samples for 1 minute at 10000 x g.
- Collect supernatant.
- Remove residual silica particles by centrifugation for 30 seconds at 12000 x g.
- Transfer supernatant to a new vial.
- Store DNA at -20°C.

*Magnetic beads extraction procedure*:

- Place filter in a 2 mL Eppendorf tube.
- Add 600 μL of GITC buffer.
- Vortex samples at maximum speed for 1 minute.
- Incubate samples at room temperature for 10 minutes.
- Pulse spin samples for 10 seconds.
- Collect 600 μL of supernatant in a fresh 2 mL Eppendorf tube.
- Add 400 μL of beads (TE) and mix 6 times with a pipette.
- Add 800 μL of isopropanol.
- Incubate samples at room temperature for 1 minute.
- Put samples on magnetic rack for 5 minutes.
- Discard supernatant and add 1600 μL of isopropanol.
- Take samples of magnetic rack, vortex at maximum speed to mix.
- Put samples on magnetic rack for 5 minutes.
- Discard supernatant.
- Add 2 mL 70% ethanol.
- Take samples of magnetic rack, vortex at maximum speed to mix.
- Put samples on magnetic rack for 5 minutes.
- Discard supernatant.
- Air-dry samples for 5 minutes.
- Resuspend samples in 200 μL of dH_2_O.
- Add 20 μL of 3 M sodium acetate.
- Add 500 μL 100% ethanol.
- Precipitate DNA overnight at -20°C.
- Centrifuge samples at 14000 x g for 20 minutes.
- Discard supernatant.
- Air-dry samples for 15 minutes.
- Resuspend pellet in 200 μL of dH_2_O.
- Store DNA at -20°C.
