# Supplementary material for: Species‐level biodiversity assessment using marine environmental DNA metabarcoding requires protocol optimization and standardization
Source: Ecol Evol. 2019 Jan 15;9(3):1323–35. doi: 10.1002/ece3.4843 (PMC6374651; doi:10.1002/ece3.4843)
Supplement: Supplementary file 3 [file ECE3-9-1323-s003.docx]

Supplement: qPCR protocol.

Amplification of all four fusion primer assays (or template-specific primer sets) were carried out in 25 μL reactions, prepared with 1x Tag Gold buffer (Applied Biosystems [ABI], USA), 2 mmol/L MgCl_2_ (ABI, USA), 0.4 mg/ml BSA (Fisher Biotec, Australia), 0.25 mmol/L dNTPs (Astral Scientific, Australia), 0.4 μmol/L of each primer (Integrated DNA Technologies, Australia), 0.6 μL of 1/10,000 SYBR Green dye (Life Technologies, USA), 1 U of Taq polymerase Gold (ABI, USA) and 2 μL of DNA. qPCR conditions were an initial denaturing step at 95°C for 5 minutes; then 50 cycles of 30 seconds at 95°C, 30 seconds at 51-54°C (see annealing temperatures in Table 2), 45 seconds at 72°C; and a final extension of 10 minutes at 72°C.
