# Supplementary material for: Species‐level biodiversity assessment using marine environmental DNA metabarcoding requires protocol optimization and standardization
Source: Ecol Evol. 2019 Jan 15;9(3):1323–35. doi: 10.1002/ece3.4843 (PMC6374651; doi:10.1002/ece3.4843)
Supplement: Supplementary file 4 [file ECE3-9-1323-s004.docx]

Supplement: Ct-values.

Figure S1: The average Ct-values obtained per replicate for each of the four assays between the optimal (blue; 1.2 μm cellulose-nitrate filter and Qiagen’s DNeasy Blood & Tissue Kit) and low-performance (gold; 1.2 μm polycarbonate filter and MO BIO’s PowerMax Soil) protocol. Error bars show 95% confidence intervals. Higher Ct-values indicate lower starting template.
