# Supplementary material for: Species‐level biodiversity assessment using marine environmental DNA metabarcoding requires protocol optimization and standardization
Source: Ecol Evol. 2019 Jan 15;9(3):1323–35. doi: 10.1002/ece3.4843 (PMC6374651; doi:10.1002/ece3.4843)
Supplement: Supplementary file 5 [file ECE3-9-1323-s005.docx]

Supplement: Sequencing information.

Table S1: Information on sequencing results displaying the total number of reads after quality control, total number of OTUs, total number of detected taxa, and the number of reads in the taxonomic dataset for each metabarcoding assay and each treatment, i.e. low-performance (PCPMS; 1.2 μm polycarbonate filter and MO BIO’s PowerMax Soil) and the optimal (CNQ; 1.2 μm cellulose-nitrate filter and Qiagen’s DNeasy Blood & Tissue Kit). Percentages between brackets for # Taxa column indicate the proportion of OTUs being classified as species. Percentages between brackets for # Reads (Taxa) column indicate the proportion of reads used in the taxonomic dataset compared to the OTU dataset.

| Genetic marker | # Reads | | | # OTUs | | | # Taxa | | | # Reads | | |
| --- | --- | --- | --- | --- | --- | --- | --- | --- | --- | --- | --- | --- |
|  | CNQ | PCPMS | Total | CNQ | PCPMS | Total | CNQ | PCPMS | Total | CNQ | PCPMS | Total |
| Fish (16S) | 53,127 | 78,465 | 131,592 | 8 | 2 | 8 | 6 (75%) | 2 (100%) | 6 (75%) | 50,604 (95.3%) | 15,253 (19.4%) | 65,857 (50.0%) |
| Crustacean (16S) | 132,933 | 93,893 | 226,826 | 29 | 9 | 31 | 14 (48.3%) | 5 (55.6%) | 14 (45.2%) | 67,463 (50.8%) | 9,936 (10.6%) | 77,399 (34.1%) |
| Eukaryotes (COI) | 239,070 | 261,688 | 500,758 | 330 | 346 | 452 | 13 (3.9%) | 14 (4.0%) | 16 (3.5%) | 7,658 (3.2%) | 5,577 (2.1%) | 13,235 (2.6%) |
| Eukaryotes (18S) | 718,985 | 728,859 | 1,447,844 | 579 | 624 | 628 | 32 (5.5%) | 44 (7.1%) | 49 (7.8%) | 171,268 (24%) | 151,509 (21%) | 322,777 (22.3%) |
