# Supplementary material for: Species‐level biodiversity assessment using marine environmental DNA metabarcoding requires protocol optimization and standardization
Source: Ecol Evol. 2019 Jan 15;9(3):1323–35. doi: 10.1002/ece3.4843 (PMC6374651; doi:10.1002/ece3.4843)
Supplement: Supplementary file 6 [file ECE3-9-1323-s006.docx]

Supplement: Species list.

| BLAST ID | Assay | Optimal protocol | | | | | Low-performance protocol | | | | |
| --- | --- | --- | --- | --- | --- | --- | --- | --- | --- | --- | --- |
|  |  | CNQ  1 | CNQ  2 | CNQ  3 | CNQ  4 | CNQ  5 | PCPMS  1 | PCPMS  2 | PCPMS  3 | PCPMS  4 | PCPMS  5 |
| Aetideidae | Eukaryotes (18S) | 0 | 0 | 0 | 0 | 0 | 0 | 3 | 0 | 1 | 0 |
| Cyclopidae | Eukaryotes (18S) | 31 | 0 | 0 | 1 | 0 | 0 | 0 | 0 | 0 | 0 |
| Dactylopusiidae | Eukaryotes (18S) | 1 | 324 | 0 | 0 | 1 | 0 | 0 | 0 | 0 | 0 |
| Harpacticidae | Eukaryotes (18S) | 0 | 0 | 0 | 0 | 0 | 0 | 12 | 647 | 11 | 0 |
| Lichomolgidae | Eukaryotes (18S) | 0 | 3 | 1 | 5246 | 8881 | 7689 | 15951 | 230 | 2427 | 331 |
| Miraciidae | Eukaryotes (18S) | 0 | 2 | 0 | 0 | 222 | 0 | 5 | 1 | 0 | 0 |
| Ascidiidae | Eukaryotes (18S) | 5 | 69 | 6 | 72 | 684 | 18 | 8 | 74 | 4 | 5 |
| Cionidae | Eukaryotes (18S) | 5 | 30 | 2 | 64 | 2 | 318 | 1174 | 623 | 352 | 143 |
| Corellidae | Eukaryotes (18S) | 99 | 10 | 1 | 5 | 6 | 4 | 15 | 24 | 7 | 23 |
| Didemnidae | Eukaryotes (18S) | 0 | 0 | 0 | 3 | 2 | 2 | 6 | 1 | 0 | 0 |
| Oikopleuridae | Eukaryotes (18S) | 0 | 59 | 0 | 78 | 2 | 6 | 40 | 2 | 33 | 1 |
| Perophoridae | Eukaryotes (18S) | 0 | 0 | 0 | 0 | 0 | 20 | 472 | 3 | 15 | 0 |
| Pyuridae | Eukaryotes (18S) | 30 | 50 | 55 | 33 | 18 | 115 | 305 | 895 | 146 | 283 |
| Styelidae | Eukaryotes (18S) | 115 | 223 | 89 | 12972 | 5609 | 395 | 5568 | 9158 | 1002 | 27140 |
| Bougainvilliidae | Eukaryotes (18S) | 0 | 0 | 0 | 0 | 0 | 2 | 0 | 0 | 3 | 0 |
| Campanulariidae | Eukaryotes (18S) | 0 | 1 | 1 | 0 | 3 | 0 | 0 | 0 | 0 | 0 |
| Mitrocomidae | Eukaryotes (18S) | 0 | 0 | 0 | 0 | 0 | 0 | 0 | 5 | 1 | 0 |
| Plumulariidae | Eukaryotes (18S) | 9 | 15 | 4 | 10 | 8 | 5 | 26 | 0 | 12 | 28 |
| Cladophoraceae | Eukaryotes (18S) | 0 | 0 | 0 | 0 | 0 | 0 | 1 | 5 | 0 | 1 |
| Kornmanniaceae | Eukaryotes (18S) | 1 | 0 | 0 | 0 | 19 | 0 | 0 | 0 | 0 | 0 |
| Posidoniaceae | Eukaryotes (18S) | 0 | 3 | 0 | 0 | 23 | 1 | 0 | 0 | 3 | 0 |
| Sargassaceae | Eukaryotes (18S) | 0 | 15 | 0 | 157 | 755 | 7 | 31 | 5 | 46 | 8 |
| Ulvaceae | Eukaryotes (18S) | 69 | 257 | 62 | 44 | 123 | 138 | 586 | 307 | 269 | 638 |
| Zosteraceae | Eukaryotes (18S) | 0 | 0 | 0 | 0 | 0 | 1 | 0 | 1 | 0 | 1 |
| Batillariidae | Eukaryotes (18S) | 0 | 0 | 0 | 0 | 0 | 6 | 3 | 0 | 6 | 1 |
| Ostreidae | Eukaryotes (18S) | 761 | 1739 | 81460 | 44645 | 2271 | 1905 | 414 | 1123 | 3304 | 974 |
| Anticomidae | Eukaryotes (18S) | 0 | 0 | 0 | 0 | 0 | 0 | 5 | 445 | 4 | 0 |
| Chromadoridae | Eukaryotes (18S) | 0 | 2 | 0 | 413 | 555 | 7426 | 6920 | 2880 | 33301 | 2688 |
| Cephalothricidae | Eukaryotes (18S) | 0 | 0 | 0 | 0 | 0 | 0 | 0 | 0 | 1 | 3 |
| Lecithasteridae | Eukaryotes (18S) | 0 | 0 | 0 | 0 | 0 | 1 | 225 | 0 | 0 | 158 |
| Cirratulidae | Eukaryotes (18S) | 0 | 0 | 0 | 0 | 0 | 0 | 4 | 4 | 0 | 1 |
| Dinophilidae | Eukaryotes (18S) | 0 | 0 | 0 | 0 | 0 | 2 | 4 | 0 | 0 | 0 |
| Polynoidae | Eukaryotes (18S) | 127 | 4 | 183 | 27 | 0 | 84 | 185 | 3641 | 35 | 0 |
| Sabellidae | Eukaryotes (18S) | 1 | 57 | 2 | 29 | 71 | 1 | 1 | 0 | 0 | 0 |
| Serpulidae | Eukaryotes (18S) | 0 | 4 | 0 | 1 | 0 | 0 | 2 | 0 | 38 | 0 |
| Syllidae | Eukaryotes (18S) | 36 | 185 | 6 | 3 | 33 | 12 | 163 | 6 | 17 | 135 |
| BLAST ID | Assay | Optimal protocol | | | | | Low-performance protocol | | | | |
|  |  | CNQ  1 | CNQ  2 | CNQ  3 | CNQ  4 | CNQ  5 | PCPMS  1 | PCPMS  2 | PCPMS  3 | PCPMS  4 | PCPMS  5 |
| Terebellidae | Eukaryotes (18S) | 1 | 2 | 4 | 2 | 4 | 2 | 17 | 22 | 5 | 8 |
| Corallinaceae | Eukaryotes (18S) | 0 | 0 | 0 | 0 | 0 | 1 | 0 | 10 | 0 | 0 |
| Lomentariaceae | Eukaryotes (18S) | 0 | 0 | 0 | 0 | 0 | 3 | 17 | 9 | 9 | 13 |
| Rhodomelaceae | Eukaryotes (18S) | 0 | 1 | 1 | 0 | 4 | 71 | 120 | 4471 | 171 | 40 |
| Schizymeniaceae | Eukaryotes (18S) | 0 | 0 | 0 | 0 | 0 | 1 | 1 | 0 | 3 | 1 |
| Callyspongiidae | Eukaryotes (18S) | 8 | 17 | 21 | 17 | 3 | 6 | 21 | 29 | 3 | 20 |
| Chalinidae | Eukaryotes (18S) | 29 | 37 | 29 | 21 | 39 | 35 | 29 | 6 | 44 | 48 |
| Clathrinidae | Eukaryotes (18S) | 0 | 0 | 0 | 0 | 0 | 1 | 2 | 1 | 5 | 6 |
| Dictyodendrillidae | Eukaryotes (18S) | 13 | 10 | 13 | 4 | 15 | 23 | 26 | 107 | 18 | 28 |
| Dysideidae | Eukaryotes (18S) | 41 | 35 | 27 | 23 | 33 | 72 | 87 | 37 | 81 | 100 |
| Halichondriidae | Eukaryotes (18S) | 381 | 651 | 127 | 158 | 160 | 180 | 637 | 156 | 148 | 555 |
| Irciniidae | Eukaryotes (18S) | 6 | 5 | 5 | 2 | 7 | 4 | 6 | 5 | 12 | 9 |
| Mycalidae | Eukaryotes (18S) | 0 | 0 | 0 | 1 | 6 | 0 | 0 | 0 | 0 | 0 |
| *Acartia* | Eukaryotes (COI) | 171 | 3 | 5307 | 0 | 749 | 680 | 86 | 805 | 750 | 1071 |
| *Bugula* | Eukaryotes (COI) | 0 | 0 | 0 | 0 | 0 | 0 | 0 | 3 | 5 | 2 |
| *Ascidia* | Eukaryotes (COI) | 0 | 3 | 0 | 1 | 0 | 0 | 3 | 0 | 1 | 1 |
| *Thyrsites* | Eukaryotes (COI) | 0 | 2 | 0 | 7 | 4 | 0 | 5 | 2 | 2 | 9 |
| *Gonothyraea* | Eukaryotes (COI) | 0 | 1 | 0 | 0 | 2 | 0 | 2 | 0 | 0 | 4 |
| *Plumularia* | Eukaryotes (COI) | 4 | 0 | 7 | 2 | 2 | 0 | 0 | 0 | 1 | 1 |
| *Hecatonema* | Eukaryotes (COI) | 0 | 1 | 0 | 1 | 3 | 0 | 0 | 0 | 0 | 0 |
| *Macrocystis* | Eukaryotes (COI) | 1 | 0 | 0 | 1 | 0 | 3 | 1 | 0 | 1 | 0 |
| *Microspongium* | Eukaryotes (COI) | 0 | 0 | 0 | 0 | 0 | 1 | 0 | 0 | 0 | 3 |
| *Undaria* | Eukaryotes (COI) | 0 | 13 | 4 | 1 | 17 | 3 | 7 | 3 | 1 | 5 |
| *Ostrea* | Eukaryotes (COI) | 191 | 61 | 412 | 154 | 134 | 28 | 38 | 1531 | 445 | 42 |
| *Corallina* | Eukaryotes (COI) | 0 | 8 | 19 | 0 | 0 | 0 | 0 | 3 | 0 | 1 |
| *Polysiphonia* | Eukaryotes (COI) | 18 | 11 | 315 | 7 | 13 | 3 | 1 | 6 | 2 | 7 |
| *Schizymenia* | Eukaryotes (COI) | 0 | 2 | 0 | 2 | 0 | 0 | 4 | 1 | 0 | 1 |
| *Callyspongia* | Eukaryotes (COI) | 0 | 0 | 0 | 0 | 0 | 0 | 1 | 1 | 1 | 0 |
| *Dendrilla* | Eukaryotes (COI) | 0 | 0 | 3 | 0 | 1 | 0 | 0 | 0 | 0 | 0 |
| *Elminius modestus* | Crustacean (16S) | 2 | 0 | 6324 | 0 | 0 | 0 | 0 | 0 | 0 | 0 |
| *Halicarcinus varius* | Crustacean (16S) | 1 | 21 | 8 | 12 | 2 | 0 | 0 | 0 | 0 | 0 |
| *Hemigrapsus sexdentatus* | Crustacean (16S) | 0 | 1 | 0 | 0 | 1 | 0 | 0 | 0 | 0 | 0 |
| *Macrophthalmus hirtipes* | Crustacean (16S) | 4278 | 12953 | 5700 | 4 | 12417 | 0 | 0 | 0 | 0 | 0 |
| *Nyctiphanes australis* | Crustacean (16S) | 489 | 2479 | 4327 | 4272 | 2567 | 0 | 0 | 0 | 1 | 1 |
| *Bugula neritina* | Crustacean (16S) | 8 | 0 | 211 | 0 | 0 | 34 | 0 | 0 | 1 | 0 |
| *Calloporina angustipora* | Crustacean (16S) | 0 | 0 | 8 | 212 | 0 | 0 | 0 | 0 | 0 | 0 |
| *Chaperiopsis cervicornis* | Crustacean (16S) | 0 | 432 | 398 | 0 | 1 | 0 | 0 | 0 | 0 | 0 |
| *Cryptosula pallasiana* | Crustacean (16S) | 0 | 0 | 6454 | 25 | 6 | 0 | 0 | 0 | 0 | 0 |
| *Scrupocellaria maderensis* | Crustacean (16S) | 99 | 0 | 0 | 0 | 90 | 0 | 0 | 0 | 0 | 0 |
| BLAST ID | Assay | Optimal protocol | | | | | Low-performance protocol | | | | |
|  |  | CNQ  1 | CNQ  2 | CNQ  3 | CNQ  4 | CNQ  5 | PCPMS  1 | PCPMS  2 | PCPMS  3 | PCPMS  4 | PCPMS  5 |
| *Watersipora subtorquata* | Crustacean (16S) | 622 | 114 | 0 | 1 | 47 | 0 | 0 | 0 | 0 | 0 |
| *Plumularia pulchella* | Crustacean (16S) | 32 | 59 | 58 | 50 | 2 | 0 | 11 | 0 | 101 | 0 |
| *Plumularia setacea* | Crustacean (16S) | 68 | 6 | 36 | 193 | 103 | 1707 | 1 | 0 | 0 | 0 |
| *Plumularia setaceoides* | Crustacean (16S) | 688 | 67 | 264 | 1130 | 121 | 4399 | 796 | 72 | 1450 | 1362 |
| *Aldrichetta forsteri* | Fish (16S) | 391 | 575 | 0 | 0 | 354 | 0 | 0 | 0 | 0 | 0 |
| *Forsterygion lapillum* | Fish (16S) | 1172 | 1966 | 837 | 3592 | 1960 | 0 | 0 | 0 | 0 | 0 |
| *Oncorhynchus tsawytscha* | Fish (16S) | 667 | 0 | 0 | 0 | 1152 | 0 | 0 | 0 | 0 | 0 |
| *Sprattus antipodum* | Fish (16S) | 15 | 4780 | 0 | 0 | 1 | 0 | 0 | 0 | 0 | 0 |
| *Sprattus muelleri* | Fish (16S) | 2105 | 1166 | 4460 | 2410 | 348 | 0 | 1 | 1 | 0 | 15248 |
| *Thyrsites atun* | Fish (16S) | 3152 | 4280 | 1218 | 5857 | 8146 | 0 | 1 | 0 | 1 | 1 |
